# Surgical Treatment of Anomalous Origin of Right Coronary Artery in a Patient with Mitral Stenosis

**DOI:** 10.3889/oamjms.2016.002

**Published:** 2015-12-24

**Authors:** Ali Refatllari, Ermal Likaj, Selman Dumani, Endri Hasimi, Artan Goda

**Affiliations:** *Department of Cardiovascular Surgery, University Hospital Centre “Mother Theresa”, Rruga e Dibres, N. 370, Tirana, Albania*

**Keywords:** Coronary anomalies, right coronary artery, bypass grafting, mitral stenosis, surgical technique

## Abstract

**BACKGROUND:**

An anomalous origin of the right coronary artery is rarely observed, with a reported incidence between 0.026% and 0.25%. This condition is often completely asymptomatic and is found incidentally during angiographic evaluation for other cardiac diseases. However some patients present with exertion angina or sudden death. Surgical treatment in patients with anomalous RCA is still controversial. Treatment can be conservative, angioplasty or surgery.

**CASE PRESENTATION:**

A 59-year-old man was admitted with severe mitral stenosis. He complained exertion and rest dyspnea, NYHA III class. He had sequels of embolic stroke, results of left atrial thrombus. Echocardiography showed calcified severe mitral stenosis with mitral orifice area of 1.1 square centimeters with PSPAP 60 mmHg and normal LV function. Routine coronary angiography before surgery showed aberrant origin of RCA from the left sinus of Valsalva with 90% stenosis at his origin. Multi-slice computed tomography proved the diagnosis of anomalous RCA arising from the left sinus of Valsalva and taking an inter-arterial course between the aorta and pulmonary artery. The patient underwent mitral valve replacement with mechanical St. Jude prosthesis No 29 and saphenous vein graft to RCA. We chose by-pass grafting techniques because after aortotomy, RCA was too close to LMCA, intramural course was too short and stenosis of RCA was outside of aortic wall. The patient’s perioperative course was without complications and patient was discharged on the seventh postoperative day.

**CONCLUSION:**

Correction of anomalous of the origin of right coronary artery is mandatory in cases where patient has to be operated for other cardiac causes.

## Introduction

Anomalous aortic origin of the coronary artery (AAOCA) is rarely observed, with a reported incidence between 0.026% and 0.25%. This condition is often completely asymptomatic and is found incidentally during angiographic evaluation for other cardiac diseases. However, some patients present with symptoms which may include chest pain, syncope, myocardial infarction, or sudden death [1-3]. AAOCA is the second most common cause of sudden death in young athletes, accounting for approximately 10% of such events [4, 5]. Surgical repair of AAOCA is safe and extremely successful in eliminating symptoms of myocardial ischemia [6].

We report here the first case in Albania with AAOCA associated with mitral stenosis diagnosed and treated surgically at our department.

## Case presentation

A 59-year-old man was admitted with severe mitral stenosis. He complained exertion and rest dyspnea, NYHA III class. No chest pain or other ischemic symptoms were seen. He had sequels of embolic stroke, results of left atrial thrombus. ECG showed atrial fibrillation and non-specific ST changes.

**Figure 1:**
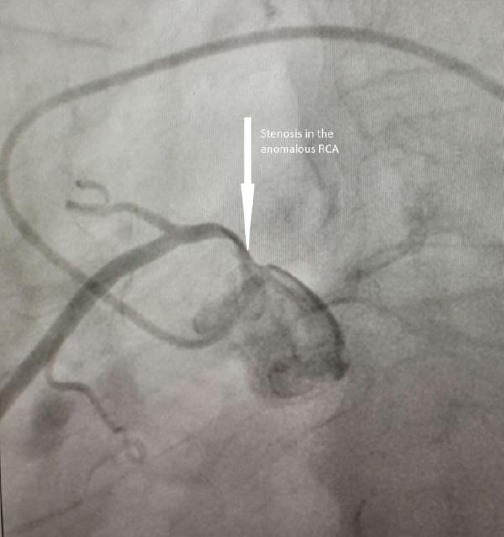
*Coronary angiography shows anomalous origin of right coronary artery from left sinus and 75-90% stenosis at its origin*.

Echocardiography showed calcified severe mitral stenosis with mitral orifice area of 1.1 cm^2^ with PASP 60 mm Hg and a normal LV function. Routine coronary angiography before surgery showed aberrant origin of RCA from the left sinus of Valsalva with 90% stenosis at his origin as seen in Figure 1. Multi-slice computed tomography proved the diagnosis of anomalous RCA arising from the left sinus of Valsalva and taking an inter-arterial course between the aorta and pulmonary artery. RCA has 90% stenosis at his origin but outside of aortic wall.

The patient underwent open heart surgery with cardiopulmonary bypass machine. After median sternotomy it was seen that right coronary artery origin was in left coronary sinus of Valsalva and the artery passed between the aorta and the pulmonary artery. After aortic clamping and mitral valve replacement with mechanical St. Jude mechanical prosthesis No 29, the aorta was divided transversally.

**Figure 2:**
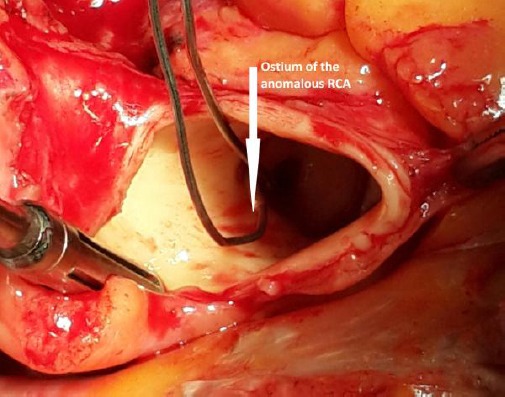
*Intraoperative images of both ostia of the coronary arteries*.

It was visualized the right coronary ostium arising from left sinus of Valsalva, close to left main ostium and its inter-arterial course. We decided to perform right coronary bypass grafting with saphenous vein to RCA because the ostium of right coronary artery was small, intramural course was too short and a 90% stenosis of RCA was outside of aortic wall. We judged that it was not the case to perform unroofing techniques. The patient’s perioperative course was without complications and patient was discharged on the seventh postoperative day.

**Figure 3:**
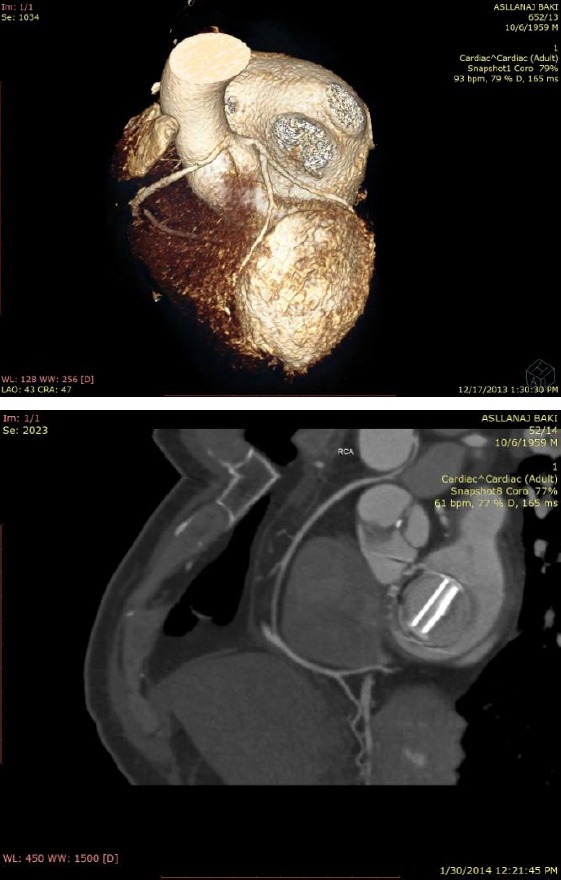
*CT scan and 3D reconstruction of the patient before (up) and after surgery (down)*.

## Discussion

Anomalous origin of the coronary artery from the opposite coronary sinus of Valsalva occurs with a very low incidence in the general population, but there is a high risk of sudden death due to myocardial ischemia and the resultant arrhythmias associated with them. Various mechanisms have been postulated to cause ischemia, including: origin in an acute angle resulting in a slit-like orifice and kinking or occlusion caused by the angulation at the point of coronary artery, coronary spasm resulting from its torsion movement, mechanical compression of the coronary artery between the pulmonary and aortic trunks during physical exertion [7-9]. The majority of these complications may be exacerbated during or immediately after exercise, as exercise leads to compression of coronary arteries as well as increasing the pre-existing angulation of the proximal portion of anomalous vessel.

Patients with AAOCA are typically asymptomatic. The diagnosis is often made as an incidental finding. Symptomatic patients complain of exertional syncope, chest pain, or palpitations [10]. The physical exam, ECG and exercise stress testing are generally unremarkable. Because coronary angiography has a significant false negative rate [11], other imaging modalities are frequently employed. In particular, multi-detector computed tomography (CT) scanners and magnetic resonance angiography (MRA) now provide excellent spatial resolution allowing visualization of the coronary anatomy [10].

The choice of treatment for this coronary anomaly is controversial, medical or surgery, with most of surgeons advocating revascularization in all of cases with inter-arterial course of anomalous coronary artery [12, 13]. Most of the authors think that in symptomatic patients, without question, surgery is the best choice. For asymptomatic patients, if they have an anomalous left coronary with an intramural route, they are offered surgery. For those with a anomalous right coronary artery arising from the left sinus of Valsalva, it is a very difficult question. The level of physical activity of these patients and the dominance of their coronary artery, also play a role in decision making. A right coronary artery that has a large posterior descending artery has a large amount of myocardium at risk, and especially in young people’s playing basketball even though they are asymptomatic, they are more likely to get operated on [14].

Our patient was free of ischemic symptoms, but he had to be operated for severe mitral stenosis and had a 90% stenosis of proximal RCA in the course of intramural anterior to the aorta (Fig. 1). No sign of atherosclerotic changes was seen in coronary angiography.

There are multiple surgical options for treating AAOCA. Bypass grafting was used initially but early graft failure was reported [15, 16]. Some authors explain that the early failure is due to the steal phenomenon at high levels of exertion [17] or competitive flow from patent native vessels contributing to graft thrombosis [18, 19]. For this reason bypass grafting has been used less frequently [10]. Other approaches include direct implantation of the anomalous artery [20], patch augmentation [21, 22] or pulmonary artery translocation to reduce the risk of compression of the anomalous vessel as it transverses between the aorta and the pulmonary artery [9, 23].

More recently, unroofing the anomalous vessel along its intramural segment has become the preferred management option [9, 24, 25]. This procedure, first reported by Mustafa in 1981, creates a neo-orifice at the anatomically correct sinus [26]. The advantages of unroofing are elimination the intramural segment and avoidanceof an oblique angle of take-off of the vessel.

We decided to perform CABG with saphenous graft in our patient for some reasons:

- there was a right coronary artery stenosis 90% outside of aortic wall, without signs of atherosclerosis but not spasm of coronary artery, because during the angiography procedure it was not released after nitrite injection;

- the right coronary ostium was small and too close to the ostium of left main coronary artery and intramural course was too short; and

- the right mammary artery was very small.

We conclude that correction of the anomalous origin of RCA is mandatory in cases where patient has to be operated for other cardiac causes.
